# The Role of p38 MAPK and Its Substrates in Neuronal Plasticity and Neurodegenerative Disease

**DOI:** 10.1155/2012/649079

**Published:** 2012-06-25

**Authors:** Sônia A. L. Corrêa, Katherine L. Eales

**Affiliations:** School of Life Sciences, The University of Warwick, Coventry CV4 7AL, UK

## Abstract

A significant amount of evidence suggests that the p38-mitogen-activated protein kinase (MAPK) signalling cascade plays a crucial role in synaptic plasticity and in neurodegenerative diseases. In this review we will discuss the cellular localisation and activation of p38 MAPK and the recent advances on the molecular and cellular mechanisms of its substrates: MAPKAPK 2 (MK2) and tau protein. In particular we will focus our attention on the understanding of the p38 MAPK-MK2 and p38 MAPK-tau activation axis in controlling neuroinflammation, actin remodelling and tau hyperphosphorylation, processes that are thought to be involved in normal ageing as well as in neurodegenerative diseases. We will also give some insight into how elucidating the precise role of p38 MAPK-MK2 and p38 MAPK-tau signalling cascades may help to identify novel therapeutic targets to slow down the symptoms observed in neurodegenerative diseases such as Alzheimer's and Parkinson's disease.

## 1. Introduction

The MAPKs are a specific class of serine/threonine kinases which respond to extracellular signals such as growth factors, mitogens, and cellular stress and mediate proliferation, differentiation, and cell survival in mammalian cells. There are 4 distinct groups of MAPKs within mammalian cells: the extracellular signal-related kinases (ERKs), the c-jun N-terminal kinases (JNKs), the atypical MAPKs (ERK3, ERK5, and ERK8), and the p38 MAPKs [1]. The p38 MAPKs are described as stress-activated protein kinases as they are primarily activated through extracellular stresses and cytokines and consequently have been extensively studied in the field of inflammation. There are, however, numerous additional roles of p38 MAPK which are becoming of interest, including the role that the p38 MAPK signalling pathway plays in neuronal function such as synaptic plasticity and neurodegenerative disease.

In the present paper we will give an overview of p38 MAPK localisation, activation, and the functional role of this signalling cascade in the mammalian brain, especially the activation of the p38 MAPK cascade during synaptic plasticity in the hippocampus. Although p38 MAPK isoforms have been shown to be highly expressed in the brain, only a handful of brain-specific substrates for p38 MAPK have been characterised *in vivo. *In this paper we will especially focus our attention on the role of two p38 MAPK substrates in neurons: MAPK-activated protein kinase 2 (MAPKAPK-2, also known as MK2) and tau protein. The involvement of the p38 MAPK-MK2 and p38 MAPK-tau signalling cascades in neuroinflammation, actin remodeling, and tau hyperphosphorylation in neurodegenerative diseases will also be discussed. Highlighting the functional role of specific p38 MAPK substrates in neurodegenerative disease will be of particular importance as these could be potential signalling targets which could be exploited therapeutically to slow cognitive decline occurring in normal ageing and in neurodegenerative disease.

## 2. Localisation and Activation of the p38 MAPK Signalling Cascade in Mammalian Cells

**Figure 1:**
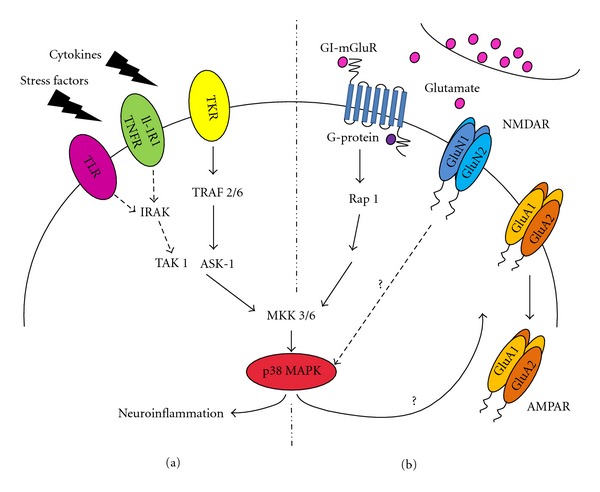
Signalling pathways leading to the activation of p38 MAPK in neurons. (a) Inflammatory cytokines bind to specific receptors at the cell surface, which initiate a cascade of events promoting the activation of interleukin-1 receptor-associated kinase (IRAK), TNF receptor-associated factor (TRAF) 2/6 leading to the activation of MKKKs (TAK 1, ASK-1), and subsequently phosphorylation of MKK3 and MKK6, the upstream activators of p38 MAPK. (b) Release of glutamate from the presynaptic terminal can also activate p38 MAPK via a similar route. Binding of glutamate by the postsynaptic GI-mGluR receptors causes the activation of G-proteins, which promote the exchange of GDP with GTP of Rap 1. Rap 1 then initiates a cascade leading to the phosphorylation of MKK3/6 and p38 MAPK. The steps linking p38 MAPK activation to the internalisation of AMPA receptor (AMPAR) subunits observed during mGluR induced long-term depression are not yet known. Reports have suggested that binding of glutamate to NMDA receptors (NMDARs) also activates p38 MAPK. However, the molecular mechanism linking NMDAR activation to p38 MAPK phosphorylation is not yet known. The activated p38 MAPK signalling cascade has been shown to regulate AMPAR trafficking; however no substrate for this regulation has been described.

The cascade of events leading to p38 MAPK activation is highly conserved throughout mammalian tissues including neuronal cells (Figures 1(a) and 1(b)). Similar to other MAPKs, the p38 MAPK enzyme is activated by dual phosphorylation of the threonine (Thr) and tyrosine (Tyr) residues in the Thr-Gly-Tyr (TGY) motif situated within the kinase activation loop. Dual phosphorylation at Thr-180 and Tyr-182 residues, by either MAP kinase kinase 3 (MKK3) or MAP kinase kinase 6 (MKK6), induces global conformational reorganisations that modify the alignment of the C- and N-terminal domains of p38 MAPK, consequently permitting the binding of ATP and the desired substrate [2]. The subcellular localisation of the p38 MAPK activators MKK3 and MKK6 has been shown using an antibody against a specific peptide present in either MKK3 or MKK6 in addition to overexpression of wild-type MKK3 and MKK6-FLAG-tagged proteins. These *in vitro* experiments in 293T HEK cells showed a similar distribution profile for both the endogenous and exogenous MKK3 and MKK6 proteins in that they are both nuclear and cytoplasmically localised within the cell [3]. The MAP kinase kinase kinases (MKKKs), which are the upstream activators of the MKKs, have been shown in HeLa cells to be activated and localised at the plasma membrane and in the cytoplasm [4]. The subcellular distribution of the upstream activators is therefore consistent with their ability to activate p38 MAPK located either in the cytoplasm as well as in the nucleus. Indeed in resting cells, including cultured hippocampal neurons, p38 MAPK protein is distributed throughout the cytoplasm and nucleus (SAL Corrêa; unpublished data, [1]).

Mammalian cells are known to express four different genes encoding p38 MAPK isoforms (p38*α*, p38*β*, p38*γ*, p38*δ*) which retain a high sequence homology between each other; p38*α* is 75% identical to p38*β* and shares 62% and 61% of identical protein sequence with p38*γ* and p38*δ*, respectively. In addition, p38*γ* shares around 70% of identical sequence with the p38*δ* isoform. The four p38 MAPKs isoforms have been shown to be widely expressed in different tissues and selectively initiate downstream responses by activating a wide range of specifically selected substrates [1, 5, 6]. Accordingly, the diversity in localisation of p38 MAPK isoforms has been implicated in a wide range of physiological processes (reviewed elsewhere [7]). The proteins that compose the p38 MAPK signalling cascade were also found to be highly expressed in mature neurons. More exciting still was the fact that MAPKs, including the p38 MAPK cascade, were found to be stimulated by glutamate receptor activation [8, 9]. In the adult mouse brain the four isoforms of p38 (*α*, *β*, *γ*, *δ*) have been shown to be expressed in tissue from the whole brain, cerebellum, and cortex using immunoblotting techniques [10]. p38*α* and p38*β* isoforms were also reported to be localised in several regions of the brain including the cerebral cortex and the hippocampus in adult mouse brain tissue using immunohistochemistry techniques [11]. Both p38*α* and p38*β* isoforms are diversely distributed within cell types and cellular compartments. Generally, throughout the brain, p38*α* is predominately expressed in neuronal cells whereas p38*β* is highly expressed in both neuronal and glial cells [11]. Diversity in isoform expression is further observed with regards to the subcellular localisation of p38*α* and p38*β* in CA1 hippocampal neurons, with p38*α* being widely distributed throughout the neuronal compartments including dendrites, cytoplasm and nucleus, and p38*β* preferentially localised in the nucleus [11]. The MKK activators MKK3/6, and additionally MKK4, are all expressed in the brain and are highly selective for the p38 MAPKs [5]. MKK6 has been shown to activate all p38 MAPK isoforms and along with MKK3 are the main activators of p38*α* which, together with p38*β*, are the most abundant isoforms of p38 MAPK to be expressed in the brain [11]. Throughout this paper only the activation of p38*α* and p38*β* isoforms will be discussed mainly due to the fact that the p38 MAPK inhibitors, SB203580 and SB202190, can specifically block the activity of the p38*α* and p38*β* and do not inhibit p38*γ* and p38*δ* isoforms [12] and also due to the fact that only p38*α* and p38*β* isoforms can phosphorylate and activate MK2.

Activation of p38 MAPK in microglia, astrocytes, and neurons can all be induced through osmotic stress and the release of cytokines such as tumour-necrosis-factor- (TNF-) *α* and interleukin (IL) 1-*α*/*β*, which activate the tumour necrosis factor receptor (TNFR) and interleukin-1 receptor (IL-1R), respectively. Interestingly, it has been shown that cultured mice astrocytes stimulated with TNF-*α* activated the p38 MAPK signalling cascade specifically through the activation of TNFR-1 and not TNFR-2, which is contrary to what has been shown in other cell types [13]. In rat cerebral microglial culture, it has been observed that p38 MAPK activation, and the subsequent production of cytokines, can be induced upon incubation with extracellular heat-shock proteins (Hsps) Hsp90, Hsp70, and Hsp32 [14]. This discovery is appealing since Hsps have been associated with a physiological protective mechanism in neurodegenerative diseases through regulating misfolded proteins and protein aggregates, like that of tau in Alzheimer's disease (AD) and *α*-synuclein in Parkinson's disease (PD). These Hsps can act as chaperones to prevent aberrant interactions between the misfolded protein aggregates and other cellular proteins and assist in reducing the accumulation of toxic oligomers in the cell by targeting the misfolded proteins for degradation [15]. Since Hsps have also been shown to induce microglial activation and downstream release of cytokines, then it can be speculated that they are involved in a neuroprotective mechanism or, if the glial cells become overstimulated, cause neuroinflammation as a result of the increase in release of inflammatory mediators. However a considerable amount of research is needed to further understand the precise role of Hsps in neurodegeneration. Additionally, it has been demonstrated that Hsp activation of the Toll-like receptor 4 (TLR4) pathway results in phosphorylation of p38 MAPK as evidence shows that there was a noticeable reduction in p38 MAPK phosphorylation and suppression of microglial release of cytokines IL-6 and TNF-*α* in the TLR4 mutant mouse [14]. 

## 3. Synaptic Plasticity and the Requirement of the p38 MAPK Signalling Cascade

Exchange of information between neurons in the central nervous system (CNS) occurs at synapses. Excitatory synapses are composed of several specialised domains including the presynaptic terminal that releases neurotransmitters and the juxtaposed postsynaptic density containing a highly dense agglomerate of proteins including (N-methyl-d-aspartate) ionotropic glutamate receptors (NMDARs) and (*α*-amino-3-hydroxy-5-methyl-4-isoxazolepropionic acid)-type glutamate receptors (AMPARs). The activity in the postsynaptic neuron is never a direct translation of the activity of the presynaptic neuron as the synaptic strength of these connections is constantly changing. This modulation in activity at glutamatergic synapses is referred to as synaptic plasticity and can often persist over long periods of time. These long-term processes are thought to be crucial in initiating the cellular changes that underlie learning and memory [16, 17]. Long-term enhancement in the strength of synaptic transmission is referred to as long-term potentiation (LTP), whereas long-term decrease in the strength of synaptic transmission is named long-term depression (LTD). A well-characterised form of LTP requires the activation of postsynaptic NMDARs which leads to the influx of calcium through the NMDAR channel. The influx of calcium initiates a cascade of events leading to the insertion of AMPAR subunits into the postsynaptic density and results in a rapid and sustained increase in synaptically evoked excitatory postsynaptic potentials (EPSPs) [16, 17]. Conversely, LTD involves either ionotropic receptors induced through the activation of NMDARs (NMDAR-LTD) or group I metabotropic glutamate receptors (GI-mGluR-LTD) [18, 19]. Activation of NMDAR or GI-mGluR triggers a diversity of signalling cascades which results in a rapid and sustained decrease in synaptically evoked EPSPs. LTP and LTD are experimentally induced in the hippocampus and over the last four decades the electrophysiological properties as well as molecular mechanisms underlying these processes have been extensively studied [16, 17]. A key step in elucidating the mechanisms underlying several forms of synaptic plasticity was the discovery that AMPAR subunits are rapidly trafficked in and out of the postsynaptic density [16, 18]. Four subunits of AMPAR, named GluA1-4, are expressed in hippocampal neurons. GluA1-4 subunits can associate in different combinations to form ion-gated channels with diverse functional properties [16, 17]. Consequently, the major challenge facing scientists investigating the regulation of AMPAR trafficking following induction of synaptic plasticity is to map the proteins involved in the cascade of events linking the activation of a specific subclass of glutamate receptors such as NMDARs or GI-mGluRs and the subsequent trafficking of AMPARs subunits. Naturally, this is a very daunting task as release of glutamate at the synaptic shaft activates distinct postsynaptic receptors and consequently several signalling pathways are stimulated simultaneously (Figure 1(b)). However, with the development of potent specific inhibitors for distinct kinase families including MAPK signalling cascades, strong evidence has emerged suggesting the requirement of MAPKs in synaptic plasticity in the hippocampus [9, 18, 20–22]. 

Accordingly, the requirement of the p38 MAPK signalling cascade in the induction of synaptic plasticity has been well characterised [9, 18] and there is a general consensus showing that p38 MAPK is required in the induction of mGluR-induced LTD [20, 23] and NMDAR-induced LTD [18]. Several studies have characterised the molecular mechanism involving the p38 MAPK cascade in GI-mGluR-dependent LTD [20, 23]. GI-mGluR-LTD involves the activation of G_q_-type G proteins which results in G*βγ* release. The subsequent activation of the small GTPase Rap 1 then activates MKKKs and MKK3/6, which activate p38 MAPK (Figure 1(b)) [23]. Although it is known that mGluR-LTD is dependent on the activation of p38 MAPK and that endocytosis governs trafficking of the cell surface AMPA receptors GluA1 and GluA2 subunits, the steps linking p38 MAPK activation to the internalisation of GluA1 and GluA2 subunits are not yet known. Furthermore, the direct targets downstream of p38 MAPK underlying LTD have not yet been elucidated (Figure 1(b)). A well-characterised target for p38 MAPK is the MAPK-activated protein kinase (MKs) subfamily, which structure, expression, and function will be discussed in the next section. Interestingly, one important biological function of the MKs is the regulation of the actin remodelling. As p38 MAPK cascade plays an important role in the induction of LTD and that an ever-increasing number of studies have linked the trafficking of AMPA receptors underlying synaptic plasticity to morphological changes in neuronal dendritic spines [24], it is plausible to speculate that activation of p38 MAPK-MKs complex can potentially be involved in actin remodelling at spines. Dendritic spines, where the majority of the glutamatergic synapses occur, are small protrusions in which stability is maintained in a dynamic fashion by the actin cytoskeleton. The processes of actin polymerisation and depolymerisation play a crucial role in the incredibly plastic size and shape of dendritic spines of hippocampal neurons. There is strong evidence to suggest that the shift in balance between the amount of G-actin and F-actin in spines is responsible for changes in the morphological characteristics of the spines such as head volume, neck length, and number of spines. Moreover, during LTP an increase in the number and size of dendritic spines occurs in neuronal cells of the hippocampus, whereas the opposite effect is observed when LTD is induced [24].

### 3.1. p38 MAPK Substrates

Three key studies were published in 1994 which provided the first step towards understanding the functional role of the p38 MAPK signalling cascade in mammalian cells. The identification of p38*α* MAPK (named as p38 MAPK, [25]) activation residues together with the discovery of MK2 as a direct substrate of p38 MAPK provided the first insight into the molecular mechanism involved in the activation of p38 MAPK cascades [26, 27]. Since then several other substrates of p38 MAPK have been identified and characterized, for example, transcription factors that are involved in cell development, cancer and myocyte differentiation such as activating transcription factor 2 and 6 (ATF2/6), tumour suppressor protein p53, nuclear factor of activated T cells (NFAT), and myocyte enhancer factors (MEF2A and MEF2C), respectively. Furthermore, proteins also involved in cell development, cancer and myocyte differentiation such as Cdc25, C/EBP homologous protein (CHOP), and kinases such as p38 activated/regulated protein kinase (PRAK) and mitogen- and stress-activated kinase (MSK1) have all be identified as p38 MAPK direct substrates (For review see [1, 28]). However, in this paper we want to focus our attention on proteins that are activated only by p38 MAPK and on proteins that play a role in regulating neuronal processes such as synaptic plasticity and neurodegenerative disease. 

Microtubule-associated protein tau, like MK2, has been shown to be phosphorylated by p38 MAPK in neurons and is therefore of interest in neuronal processes. In the following sections we will describe the localisation, activation, and, where possible, the physiological role of these two substrates of p38 MAPK, MK2 and tau, and their role in actin remodelling. While the role of the p38 MAPK-MK2 cascade in actin remodelling through posttranslational modifications has not yet been studied in detail in neurons, a significant amount of information is available on the molecular mechanism by which p38 MAPK regulates neuronal tau function.

#### 3.1.1. MAPKAP Kinases (MKs)

MAPKAPK-2 (MK2) and MAPKAPK-3 (MK3) are serine/threonine kinases belonging to the MAPK-activated protein kinase subfamily that bind to and are activated specifically by the p38*α*/*β* MAPK isoforms [29, 30]. MK2 is believed to be one of the most important kinases to be activated by p38*α*/*β* MAPK due to its vital role in mediating the cellular stress and inflammatory responses. 

The MK2 enzyme is composed of a proline-rich N-terminal domain, a catalytic domain, a C-terminal domain containing an autoinhibitory A-helix (AH), the nuclear export signal (NES), the nuclear localisation signal (NLS), and the p38 MAPK-binding domain. Once activated, p38*α*/*β* phosphorylate MK2 at Thr-222 located in the activation loop, at Ser-272 located within the catalytic domain, and at Thr-334, another regulatory phosphorylation site. One of the most important characteristics of MK2 is the ability to behave as a bifunctional switch, linking kinase activation to its subcellular localisation. Upon phosphorylation at Thr-334 by p38*α*/*β* MAPK, a conformational change occurs within the MK2 structure allowing the exposure of the masked NES as well as the exposure of the substrate-binding site, permitting the translocation of the activated MK2 from the nucleus to the cytoplasm of the cell. Contrary to the regulated function of the NES, the NLS motif is active independently of the phosphorylation state of MK2 therefore permitting the kinase to shuttle between nucleus and cytoplasm [29, 30]. Although both NES and NLS domains are accessible in the kinase active state, it seems that the nuclear export signal is more effective than the import signal. Therefore, most of the shuttling of the active p38 MAPK-MK2 is cytoplasmic, which directs the active p38 MAPK-MK2 complex to be localised and to phosphorylate substrates in the cytoplasm. Regarding the subcellular localisation of MK2 in neurons, a significant increase in levels of MK2 mRNA has been reported in pyramidal cell layers of CA1 and CA3 and in the granule cell layer of the dentate gyrus regions of the hippocampus after kainic-acid-induced seizures [31]. Furthermore, high levels of endogenous MK2 protein are also observed in the hippocampus and frontal cortex of postnatal and adult mice using immunoblotting techniques (SAL Corrêa, unpublished data). 

Several proteins have been found to be phosphorylated by MKs, which implicates the role of this enzyme in a wide range of cellular functions [29]. These include interactions with heat-shock proteins (Hsps) [32, 33], the p16 subunit (p16-Arc) of the seven-member actin-related protein-2/3 complex (Arp2/3) [34], F-actin capping protein Z-interacting protein (Cap-ZIP) [35], and lymphocyte-specific protein (LSP)1 [36]. In 2011 an elegant study from Matthias Gaestel's laboratory showed that the p38 MAPK-MK2 complex plays an important role in the activation of serum-response-element- (SRE-) driven immediate early genes (IEGs) by direct phosphorylation of serum response factors (SRF) at Ser-103 [37]. These findings are very exciting as transcriptional activation of Arc/Arg3.1 (activity-regulated cytoskeleton-associated protein or activity-regulated gene 3.1), which is a neuron-specific IEG, has been shown to be dependent on SRF activation in primary cortical neuron culture [38, 39]. Arc/Arg3.1 is thought to be a key regulator of specific forms of synaptic plasticity that depend on protein synthesis [39]. Accordingly, Arc/Arg3.1 has been shown to regulate spine morphology [40], control trafficking of AMPA receptors through its interaction with the endocytic machinery [41], and is believed to regulate an endosomal pathway involved in the generation of activity-dependent amyloid-beta (A*β*) deposits [42]. However, the precise signalling cascade(s) that modulates Arc/Arg3.1 function is far from clear. All MK2 substrates described above are involved in controlling actin remodelling, which suggests that the p38 MAPK-MK2/3 signalling cascade may play an important role in the rearrangement of the actin cytoskeleton. Although several other substrates for MKs have been characterised [29], in this paper we will focus on the MK2 substrates that potentially play a physiological role in controlling actin dynamics in neurons and a pathophysiological role in neuroinflammation and stress responses. 

#### 3.1.2. Tau

Tau is a highly soluble microtubule-associated protein (MAP) in which subcellular localisation is determined by its phosphorylation status in neuronal cells. The principal function of tau is to bind and stabilise cytoskeleton microtubules (MTs) and thus tau protein is characterised by the presence of a microtubule-binding domain. This domain is comprised of multiple, highly conserved repeats of a tubulin-binding motif and it is the number of these repeats which defines the identity of each of the tau isoforms. Tau can bind to microtubules through the globular protein tubulin, which is the basic unit of microtubules. The tubulin-binding repeats within the MT-binding domain bind to specific regions of *β*-tubulin which are located on the microtubule inner surface. Additionally, the positively charged proline rich region, which is situated before the MT-binding domain, can tightly bind to the negatively charged microtubule surface [43]. These interactions therefore contribute to the stabilisation of microtubules. The ability and binding affinity of tau to interact with microtubules is regulated through post-translational modifications, mainly through phosphorylation of serine and threonine residues. This regulatory phosphorylation is tightly controlled by numerous protein phosphatases and kinases, and consequently p38 MAPK has been identified as one of the kinases involved in tau regulation [44, 45]. 

p38 MAPK can directly phosphorylate tau protein *in vitro* and *in vivo.* There are 85 putative phosphorylation sites situated on tau of which 79 are either serine (45) or threonine residues (34) [44, 46, 47]. Of these, p38 MAPK has been shown to phosphorylate Ser-46, Thr-181, Ser-202, Thr-205, Thr-212, Thr-217, Thr-231, Ser-235, Ser-356, Ser-396 and Ser-404 [44]. Tau has been shown to be predominately localised to the axonal regions of nonstimulated neurons [48]. However, when the proline-rich region of tau is hyperphosphorylated, tau is seen to be localised in the somatodendritic compartments of neurons [49–52]. Therefore, the phosphorylation status of tau is of crucial importance in determining its binding partners and consequently its functional role as a result of its differential localisation. The phosphorylation of a particular residue Ser-356 has been proposed to instigate detachment of tau from the microtubules [44, 47, 53], and since this residue has been shown to be phosphorylated by p38 MAPK then p38 MAPK could potentially be involved in the destabilisation of the microtubules. Additionally, phosphorylation of residues Thr-231 and Ser-235 by p38 MAPK has also been shown to contribute to tau detachment from microtubules [54]. This physiological role of p38 MAPK in tau phosphorylation can turn into a pathophysiological role if tau becomes hyperphosphorylated. Since this hyperphosphorylation results in increased tau detachment from microtubules, then there is an increase in the amount of soluble tau present in the neuron, which is therefore prone to self-aggregation and polymerisation, leading to the formation of tau oligomers. These oligomers combine and further aggregate to form paired helical filaments (PHFs) which then assemble to form neurofibrillary tangles (NFTs) as seen within diseases such as Alzheimer's disease. 

### 3.2. p38 MAPK in Neurodegenerative Diseases

Dysfunction within neuronal signalling pathways led to neurodegenerative diseases and the p38 MAPK signalling pathway is no exception. Irregularities in p38 MAPK signalling in neuronal cells have been linked with neuroinflammatory processes and with diseases such as Alzheimer's disease, Parkinson's disease, amyotrophic lateral sclerosis (ALS), and Pick's Disease (PiD).

#### 3.2.1. Neuroinflammation

The process of acute inflammation in mammalian tissue is one of extreme importance as it is the immediate cellular response to injury and is a defensive mechanism to prevent damage to the cell. Chronic inflammation occurs when there are persistent inflammatory stimuli that can have a damaging rather than protective effect. For example, chronic glial cell activation is seen to be increased in neurodegenerative disease [55], which will be discussed in the following section. 

**Figure 2:**
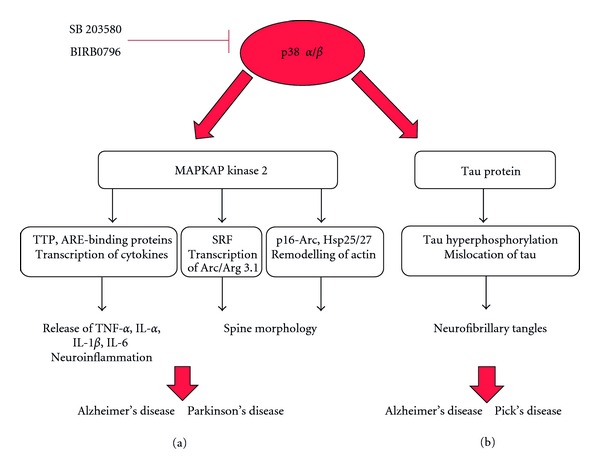
Schematic drawing illustrating the steps linking the p38 MAPK substrates to neurodegenerative disease. (a) The p38 MAPK-MK2 complex plays a role in neuroinflammation by phosphorylating AU-rich-element- (ARE-) binding proteins, such as tristetraprolin (TTP), which consequently can bind directly or indirectly to ARE sites present in TNF and other cytokine genes leading to transcription, translation, and subsequent release of mediators causing inflammation. The p38 MAPK-MK2 axis potentially plays an important role controlling dendritic spine morphology via direct activation of p16-Arc and Hsp, which are proteins involved in actin remodelling. Activity-dependent induction of p38 MAPK-MK2 axis can play an important role in the expression of the immediate early gene Arc/Arg3.1 which regulates spine morphology in neurons via activation of serum-response-factor- (SRF-) serum response element (SRE) complex. p38 MAPK-MK2 signalling cascade activation can have an effect on morphological changes observed at dendritic spines, a pattern that is observed during the development of neurodegenerative disease. (b) p38 MAPK phosphorylates tau protein at several residues. Hyperphosphorylated tau, contributes to the formation of tau oligomers. The aggregation of the tau oligomers forms the paired-helical filaments (PHFs), which then assemble together to form neurofibrillary tangles that are characteristically observed in the brain of patients suffering from Alzheimer's disease.

One of the many physiological roles of glial cells within the brain, such as astrocytes and microglia, is to protect the brain from stress and other cellular stimuli and to act as mediators in inflammation and neuroprotection. Prolonged and sustained activation of glial cells can result in an exaggerated inflammatory response and as a result cause neuronal cell death through the elevated release of proinflammatory cytokines, which have a potential neurotoxic effect, leading to increased neurodegeneration [56, 57]. The majority of studies investigating the role of p38 MAPK in mammalian cells focus on its function in the process of inflammation. It is known that p38 MAPK becomes stimulated in response to extracellular stimuli such as stress factors and cytokines [58, 59] (Figure 1(a)). Exposures of cells to stress factors/cytokines stimuli activate a number of MKKKs, for example, TGF-beta-activated kinase 1 (TAK 1) and apoptosis signal-regulated kinase 1 (ASK-1) (Figure 1(a)). The function of these kinases is to phosphorylate the downstream kinases MKK3/6, which are the known activators of p38*α*/*β* MAPK. Many p38 MAPK targets are kinases and transcription factors which are known to play a role in inflammation through the production and activation of inflammatory mediators. The p38 MAPK-MK2 complex is known to contribute to the inflammation process as it has been observed *in vivo* that MK2 knockout mice are resistant to endotoxic shock when stimulated with lipopolysaccharide LPS (Figure 2(b)) [60]. It has also been recognised that MK2 is involved in regulating the production of TNF-*α*, interleukin-6 (IL-6), interleukin-8 (IL-8), and other cytokines which all play a role in the process of inflammation [61, 62]. In addition it has been seen that MK2 expression and activation is increased in LPS- and interferon-*γ*-stimulated microglial cells, which can release inflammatory mediators, and that microglial cells cultured from MK2 knockout mice showed a decrease in the release of inflammatory cytokines [63]. This signalling is of particular interest as it has been documented that the p38 MAPK-MK2 pathway and the consequent production of inflammatory cytokines have a significant role in neurodegenerative disease with oxidative stress and persistent neuroinflammation being the primary cause for such disease (Figure 2(a)). 

#### 3.2.2. Alzheimer's Disease

Alzheimer's disease is the most common form of dementia and is becoming increasingly prevalent with an estimation that 1 in 85 people globally will be affected by 2050 [64]. The disease is typically characterised by the presence of A*β* plaques or NFTs formed from free aggregated neuronal tau within the brain. However the relationship between these structures and the symptoms of cognitive impairment and memory loss that is associated with the disease remains uncertain. Increasing evidence has shown that the stability of dendritic spines and actin remodelling may participate in the pathology of the disease. The loss of synapses is a common occurrence within postmortem tissue of AD patients [65]. Many studies and findings in animal models have linked early symptoms of AD with loss in cognition, combined with a reduced number of dendritic spines in the hippocampus [66, 67], which is one of the most affected areas of the brain in this disease. It has been shown that dendritic spines become increasing destabilised, have aberrant morphology, and are subject to degeneration as a consequence of the accumulation of toxic A*β* oligomers [68] and their direct binding with the spine head [69, 70]. The direct A*β*-oligomer-binding site is not yet known; however evidence shows these oligomers act as specific ligands to bind to or near targets on the spine surface and it has been observed that these oligomers bind to neurons from mature hippocampal cultures expressing the NMDA receptor subunits, GluN1 and GluN2B but not astrocytes and inhibitory neurons [69, 70]. Furthermore, analysis of postmortem tissue collected from AD patients has shown an increase in the amount of p38 MAPK phosphorylation associated with A*β* plaques and NFTs [71] and that mutant tau (P301L) becomes mislocated to the somatodendritic compartments of the neuron compared to healthy neurons where tau is expressed principally throughout the axon [45, 48]. 

Although the predominant function of tau protein is to assist in the stabilisation of microtubules through its binding to *β*-tubulin, it possesses additional regulatory functions. Recent interesting findings have shown that there is a significant increase in the binding affinity between hyperphosphorylated tau and F-actin *in vivo*. Experiments using brains of transgenic *Drosophila melanogaster *expressing wild-type human tau or a hyperphosphorylated mutant (R406W) form clearly demonstrated that the amount of F-actin immunoprecipitated with hyperphosphorylated tau mutant is significantly higher when compared with the amount of F-actin immunoprecipitated with wild-type tau [72–74]. Little information is available about the interaction sites between tau and actin. However it is known that the microtubule-binding domain (MTBD) is primarily involved with this association [75, 76]. Furthermore, it has been shown that the proline-rich domain of tau protein is important in assisting the actin-tau interaction as this domain alone is able to bind to F-actin and can even promote F-actin bundling [77]. 

Elucidating the precise molecular mechanism underlying the rearrangement of the actin cytoskeleton in spines is extremely important. Potentially, the physiological role of the p38 MAPK signalling cascade could be involved in the rearrangement of the actin cytoskeleton in dendritic spines through different targets. Activity-dependent induction of the p38 MAPK-MK2 axis leading to the phosphorylation of SRF in neurons can potentially trigger the activation of Arc/Arg3.1 transcription (Figure 2(a)). Given the importance of Arc/Arg3.1 protein in the molecular mechanism underlying synaptic plasticity, in regulating spine morphology and in promoting the stability of the actin network, the knowledge of the precise signalling cascade(s) controlling Arc/Arg 3.1 transcription could provide insightful information on the function of this multitalented protein and its function in neurodegenerative disease [39–42]. Activated p38 MAPK-MK2 axis could also potentially regulate Arp2/3 complex through phosphorylation of actin remodelling proteins such as p16-Arc in neurons (Figure 2(a)). p16-Arc has been shown to interact with and is phosphorylated by MK2 at Ser-77 *in vitro* [34]. Strong evidence suggests that the Arp2/3 complex is required for changes in dendritic spine morphology as it plays a key role in the formation of branched actin filamentous networks [78–80]. Another potentially important role for p38 MAPK signalling pathway in actin remodelling in neurons is via tau. p38 MAPK-dependent hyperphosphorylation of tau could induce tau mislocation from the axon to dendritic spines, where hyperphosphorylated tau is then able to bind to F-actin (Figure 2(b)) [72]. However further experimental work in neurons is needed not only to validate the p38 MAPK downstream substrates, but also to show their functional importance in remodelling dendritic spines in healthy neurons and in neurodegenerative diseases.

In addition to the aforementioned association with plaques and tangles, p38 MAPK is involved with the inflammatory response. It was shown that A*β* is able to stimulate glial cell cultures and activate p38 MAPK [81] and MK2, thus upregulating the production of inflammatory cytokines such as IL-1*β* and TNF-*α* in hippocampal extracts (Figure 2(b)) [82, 83]. It is this increased release of inflammatory mediators from overstimulation of A*β*-stimulated glial cells that can cause a neuroinflammatory and neurotoxic effect on surrounding neurons [84], contributing to the loss of neurons witnessed in neurodegenerative disease. A*β* has also been shown to stimulate microglia *in vivo,* where direct injection of A*β* into rat striatum resulted in the activation of microglia, the production of cytokines, and eventually loss of neuronal cells [85]. In AD, it has been shown that microglia accumulate at the site of A*β* deposition and actively clear such deposits [86] through their phagocytic abilities. Microglia also produce a neuroprotective inflammatory response from activation of toll-like receptors (TLRs) which can induce the production of inflammatory mediators through the MKK6-p38 MAPK-MK2 cascade, as described previously (Figures 1(a) and 2(b)). If glial overactivation occurs through A*β* plaque stimulation, then this inflammatory response can lead to neuronal cell death as observed in rat brain *in vivo* [87]. Upon the application of the novel and selective p38*α* MAPK inhibitor MW01-2-069A-SRM (069A), this increase in the production of inflammatory molecules from glial cells is blocked [82] hence highlighting the importance of p38 MAPK as a target to combat neuroinflammation and the pathological consequences that arise as a result. 

Glial-neuron interactions and the effect these interactions have on tau phosphorylation have been analysed *in vitro*. It has been demonstrated that release of proinflammatory cytokine IL-1 from activated microglia increased the levels of tau phosphorylation in neurons. These changes are partly mediated through activation of p38 MAPK as a significant increase in the levels of phospho-p38 MAPK was observed upon application of IL-1*β* in cultures of neocortical neurons and microglia [88]. Additionally, upon inhibition of p38 MAPK with SB203580, it was observed *in vitro* that IL-1*β*-induced tau phosphorylation was considerably decreased in neuronal culture [88], again highlighting the importance of p38 MAPK in cytokine release and tau phosphorylation, linking chronic glial cell activation and interactions with neurons with tau pathology in neurodegenerative disease. 

Under pathophysiological conditions, activated p38*α* has been seen to localise to areas where NFTs, amyloid plaques, and glial cells are present both within human AD brain and transgenic mouse models [71, 89, 90]. Furthermore, an increase in the activation and expression levels of one of the upstream activators of p38 MAPK, MKK6, has been observed in AD brain tissue [91] and to be colocalised with activated p38 MAPK in areas containing NFTs and plaques. Additionally, ASK-1, a specific activator of MKK6, has been shown to form an active complex with amyloid precursor protein (APP) [92], the precursor to A*β*. This complex, which forms through A*β*-peptide-induced dimerisation, suggests a connection between the aberrant processing of APP and the ASK-1-MKK6-p38 MAPK cascade which is involved in inflammation and abnormal tau phosphorylation [93]. *In vitro* activation of MKK6-p38 MAPK has led to tau phosphorylation at specific sites, the most efficient being Ser-396, which has been suggested to have a functional role in microtubule binding. Abnormal phosphorylation at Ser-396 is observed in AD brain but not in normal functioning adult brain [94]. Furthermore, it has been seen in AD hippocampal extracts taken from post-mortem human brain that MKK6 can coimmunopreciptate with phosphorylated tau protein and that APP is also able to coimmunoprecipitate with both ASK-1 and MKK6 [93]. This suggests that APP, once stimulated by A*β* peptide, can activate the upstream MKKK ASK-1 and MKK6, consequently activating p38 MAPK, which can directly phosphorylate tau protein hence linking deposition of A*β* plaques to downstream tau phosphorylation through the activation of the p38 MAPK signalling cascade under pathophysiological conditions. 

Pick's disease is another severe neurodegenerative disorder, which involves progressive dementia and aphasia through the development of Pick bodies, which are comprised of neurofibrils formed of aggregated phosphorylated tau. It is known that oxidative stress is involved in instigating Pick's disease, and since it has been highlighted that the p38 MAPK cascade is activated upon such stimuli, it may play an important role in this disease as well. It has been observed in post-mortem brain tissue that phosphorylated p38 MAPK localises to the Pick bodies which contain highly phosphorylated tau protein, and since p38 MAPK is capable of phosphorylating tau, as described above, it emphasises the importance of p38 MAPK in this disease as well as AD and other related tauopathies [95].

#### 3.2.3. Parkinson's Disease

Parkinson's disease is the second most prevalent neurodegenerative disease and around 127,000 people in the UK are currently living with the disease, which has been estimated to rise by 28% by the year 2020 [96]. PD involves the substantial loss of dopaminergic neurons from the substantia nigra and the accumulation of aggregated protein deposits, which form cytoplasmic inclusions called Lewy bodies (LBs). The aggregation of these protein dense structures subsequently causes defects in the central nervous system and severely impairs motor and cognitive abilities [97]. Mutations within 9 genes (SNCA, UCHL1, LRRK2, GIGYF2, HtrA2, PRKN, PINK1, DJ-1, and ATP13A2) [98] have been discovered and linked to PD. An important gene encodes *α*-synuclein (SNCA), which is a protein present within the LBs of PD patients and known to play a pivotal role in the development of the disease. *α*-Synuclein is highly expressed in neuronal tissue and is subject to many post-translational modifications such as phosphorylation and ubiquitination, although its physiological function is poorly characterised. It has been suggested, however, that these post-translational modifications participate in neurotoxicity [99] as aggregated *α*-synuclein is the predominant fibrillar component of the proteinaceous Lewy bodies seen in PD. Additionally, it has been observed that amplified *α*-synuclein levels are linked to an increase in neuroinflammation as it has been shown that *α*-synuclein activates p38 MAPK and other MAPKs in glial cells, a finding that is supported by the fact that extracellular *α*-synuclein released from damaged neurons interacts with microglia [100]. As described above, the activation of glial cells through the p38 MAPK-MK2 cascade consequently results in the production of inflammatory cytokines and TNF-*α*, which can induce and promote neuroinflammation as a physiological neuroprotective mechanism. If the glial cells become overactivated, however, as may occur through elevated levels of extracellular *α*-synuclein released from damaged Lewy body containing neurons, then chronic inflammation could be established and lead to neuronal cell death. 

MAPKAP kinase-2, one of p38 MAPK more prevalent substrates has also been implicated within PD, where it has been shown that MK2-deficient mice show decreased levels of neuroinflammation and loss of dopaminergic neurons within the substantia nigra after treatment with the Parkinson's inducing neurotoxin MPTP compared to MK2 wild-type mice [101]. MK2, after activation by p38 MAPK, is known to induce the transcription and release of proinflammatory molecules such as IL-1, IL-6, and TNF-*α*, thus causing neuroinflammation and potentially neuronal cell death. An investigation by Culbert et al. [63] demonstrates that if MK2 is eliminated in microglia then this neurotoxic inflammatory mechanism is reduced as the release of proinflammatory mediators is inhibited, resulting in mice that exhibit a neuroprotective phenotype therefore preventing neuronal cell death.

## 4. Concluding Remarks

Considerable progress has been made in the understanding of the functional role of the p38 MAPK signalling cascade in synaptic plasticity in the hippocampus and its potential role in neurodegenerative diseases such as AD. However, less is known regarding the role of the direct targets of p38 MAPK, such as MK2 and tau, in regulating neuroinflammation and the actin cytoskeleton in dendritic spines of neuronal cells. Growing evidence suggests that remodelling of actin at dendritic spines plays a crucial role in synaptic plasticity and therefore in cognitive processes such as learning and memory. Furthermore, recent findings in animal models have linked early symptoms of AD with loss of cognitive functions, combined with a reduced number of dendritic spines in the hippocampus. Abnormal dendritic spine morphology has also been observed in brain tissue from patients suffering from AD. Therefore, with an ageing population continuing to grow and the consequent rise in AD, elucidating the precise role of p38 MAPK-MK2 and p38 MAPK-tau signalling cascades in controlling actin remodelling becomes very important as it may identify novel targets to slow down the cognitive decline observed in normal ageing and in the early stages of neurodegenerative diseases.
